# Luteolin alleviates PCOS by inhibiting AR/STAT3/NLRP3-mediated granulosa cell pyroptosis

**DOI:** 10.1186/s13048-025-01952-4

**Published:** 2026-01-24

**Authors:** Xiaoling Ouyang, Hong Tang, Yuting Yang, Xiaobai Hao, Xiaomei Jiang, Qi Zhou, Linxia Li

**Affiliations:** 1 Departments of Gynaecology and Obstetrics, The Seventh People’s Hospital of Shanghai University of Traditional Chinese Medicine, Shanghai, 200137 China; 2 Central Laboratory, The Seventh People’s Hospital of Shanghai University of Traditional Chinese Medicine, Shanghai, 200137 China; 3 Department of Obstetrics and Gynecology, Shanghai General Hospital, Shanghai Jiao Tong University School of Medicine, 85 Wujin Road, Hongkou, Shanghai, 200080 China

**Keywords:** Polycystic ovary syndrome, Hyperandrogenemia, Chronic inflammation, Pyroptosis, Luteolin

## Abstract

**Background:**

Polycystic ovary syndrome (PCOS), a common endocrine-metabolic disorder, is driven by hyperandrogenism and chronic low-grade inflammation that impair follicular development. Granulosa cell pyroptosis is increasingly recognized as a key pathogenic mechanism in PCOS. *Luteolin* (LUT), a natural flavonoid found in many traditional medicinal plants, exhibits potent anti-inflammatory properties. However, its role in regulating granulosa cell pyroptosis within the context of PCOS has not been elucidated.

**Methods:**

We established a dehydroepiandrosterone (DHEA)-induced PCOS rat model to evaluate LUT’s therapeutic effects. Hormone and cytokine levels were measured by enzyme-linked immunosorbent assay (ELISA), while network pharmacology and molecular docking were used to predict molecular targets. In vitro, dihydrotestosterone (DHT)-treated KGN cells served as a model for granulosa cell dysfunction. Pyroptosis was assessed by Cell Counting Kit−8 (CCK-8), lactate dehydrogenase (LDH) release, and transmission electron microscopy. The expression and activation of androgen receptor (AR), Signal Transducer and Activator of Transcription 3 (STAT3), and NOD-like Receptor Pyrin domain-containing protein 3 (NLRP3) inflammasome components were analyzed by Western blot and immunohistochemistry, with their roles confirmed using specific inhibitors.

**Results:**

Luteolin (LUT) treatment alleviated hormonal imbalance and ovarian morphological abnormalities in PCOS rats. LUT suppressed STAT3 phosphorylation, pro-inflammatory cytokine expression, and NLRP3 inflammasome activation in both in vivo and in vitro models. Network pharmacology identified STAT3 as a high-affinity target of LUT (binding energy: − 8.589 kcal/mol). Mechanistically, LUT attenuated granulosa cell pyroptosis by suppressing the AR/STAT3/NLRP3 axis.

**Conclusion:**

Luteolin inhibits androgen-induced granulosa cell pyroptosis by targeting the AR/STAT3/NLRP3 signaling pathway. These findings provide a robust mechanistic basis for luteolin’s therapeutic potential in PCOS, supporting its development as a targeted therapy for this and other inflammatory reproductive disorders.

**Supplementary Information:**

The online version contains supplementary material available at 10.1186/s13048-025-01952-4.

## Introduction

Polycystic ovary syndrome (PCOS) is a prevalent and complex endocrine-metabolic disorder affecting 5–18% of reproductive-aged women worldwide. The syndrome is clinically characterized by hyperandrogenism, anovulation, and polycystic ovarian morphology. These features are underpinned by a state of chronic low-grade inflammation, which is frequently exacerbated by metabolic comorbidities such as insulin resistance and obesity [1–3]. Within its multifactorial etiology, hyperandrogenemia (HA) is recognized as a cornerstone of PCOS pathophysiology, driving follicular arrest and impairing ovarian function [4, 5].

Emerging evidence implicates granulosa cell pyroptosis—an inflammatory form of programmed cell death—as a key pathogenic mechanism in PCOS. This process is primarily mediated by the NOD-like Receptor Pyrin domain-containing protein 3 (NLRP3) inflammasome, whose activation triggers caspase-1-dependent secretion of pro-inflammatory cytokines, including Interleukin-1β (IL-1β) and Interleukin-18 (IL-18) [6–8]. Significantly, HA has been identified as a direct trigger for NLRP3 inflammasome activation in granulosa cells [9]. While these studies establish a crucial link between hyperandrogenism and inflammation, they primarily focus on the downstream inflammatory consequences rather than the upstream signaling events initiated by the androgen receptor (AR). For instance, recent literature has implicated AR in ovarian inflammation, but has not identified the direct transcriptional targets that mediate pyroptosis [9–11]. Therefore, understanding how AR signaling translates into NLRP3 expression represents a major gap in our understanding.

To fill this gap, this study proposes that Signal Transducer and Activator of Transcription 3 (STAT3) functions as the critical intermediary in this pathway. STAT3 is a well-established downstream effector of AR signaling and has also been reported to act as a direct transcriptional regulator of NLRP3 [9–13]. While both the AR-STAT3 and STAT3-NLRP3 links have been described in other contexts, our study is the first to connect them into a single, functional axis within PCOS granulosa cells. Based on this evidence, we hypothesized that HA, acting through AR, promotes STAT3 activation, which in turn transcriptionally upregulates the NLRP3 inflammasome to induce granulosa cell pyroptosis. This proposed AR/STAT3/NLRP3 signaling axis represents a novel pathogenic mechanism contributing to ovarian dysfunction in PCOS.

Luteolin (LUT), a natural flavonoid compound abundant in traditional medicinal plants such as *Lonicera japonica*, *Perilla frutescens*, and *Scutellaria baicalensis*, has long history in ethnomedicine for treating gynecological and inflammatory conditions [14–16]. Modern pharmacological studies have validated its potent antioxidant and anti-inflammatory properties. For instance, in PCOS models, LUT has demonstrated efficacy in improving insulin sensitivity and reducing oxidative stress [17–20]. Despite these promising findings, whether LUT can directly counteract PCOS-related pathology by targeting pyroptosis in granulosa cells remains unexplored.

Therefore, this study investigates whether LUT alleviates PCOS by inhibiting granulosa cell pyroptosis. By integrating network pharmacology, molecular docking, and rigorous in vivo and in vitro validation, we sought to elucidate the role of the AR/STAT3/NLRP3 axis in mediating LUT’s protective effects. This work not only provides a novel mechanistic basis for the therapeutic application of a traditional medicine-derived compound but also identifies a promising strategy for targeted intervention in PCOS.

## Materials and methods

### Animals and treatments

Twenty-three-day-old female Sprague–Dawley (SD) rats were purchased from SLAC Animals (Shanghai, China). All animal experiments were approved by the Shanghai Seventh People’s Hospital Ethics Committee (approved number: 2025-AR−003). Animals were housed under specific pathogen-free (SPF) conditions (25 ± 2 °C, 55 ± 5% humidity, 12 h light/dark cycle) with ad libitum access to standard chow and water.

After one-week acclimatization, rats were randomly assigned to four groups (*n* = 6): control, PCOS, LUT low-dose (LUT-L), and LUT high-dose (LUT-H). The sample size was selected based on previous studies using similar DHEA-induced PCOS models, which demonstrated that six animals per group provide sufficient statistical power to detect meaningful inter-group differences [21, 22].

To induce the PCOS model, rats in the PCOS, LUT-L, and LUT-H groups received daily subcutaneous injections of dehydroepiandrosterone (DHEA, 60 mg/kg in corn oil) and human chorionic gonadotropin (HCG, 1.5 IU) for 21 consecutive days. The control group received 0.9% saline. Following PCOS model establishment, a 28-day treatment period commenced. The LUT-L and LUT-H groups were administered luteolin (suspended in 0.5% CMC-Na) by gavage, while the Control and PCOS groups received the vehicle (0.5% CMC-Na). The selection of luteolin doses was based on a prior study [19].

### Vaginal smear assay

The estrous cycle of rats was monitored daily at a consistent time for 7 consecutive days using a vaginal smear assay. Vaginal secretions were collected by gently flushing the vagina with saline using a Pasteur pipette, and the sample was transferred onto a glass slide. Next, a Pasteur stain kit was used for staining. The estrous cycle stages were identified through microscopic examination of the stained cells.

### Enzyme-linked immunosorbent assay (ELISA)

Serum levels of sex hormones and inflammatory cytokines were quantified using commercially available enzyme-linked immunosorbent assay (ELISA) kits (Elabscience, Wuhan, China) according to the manufacturer’s instructions. The specific kits used were for follicle-stimulating hormone (FSH; Elabscience, E-EL-R0391), luteinizing hormone (LH; Elabscience, E-EL-R0026), testosterone (T; Elabscience, E-OSEL-R0003), estradiol (E2; Elabscience, E-OSEL-R0001), interleukin−6 (IL−6; Elabscience, E-EL-R0015), interleukin−1β (IL−1β; Elabscience, E-EL-R0012), and interleukin−18 (IL−18; Elabscience, E-EL-R0567).

### Hematoxylin and Eosin staining (H&E)

Ovarian tissues were collected and fixed in 4% paraformaldehyde for 24 h. The fixed tissues were then dehydrated in a graded ethanol series, cleared with xylene, and embedded in paraffin. The paraffin-embedded blocks were sectioned at 5 μm thickness. Finally, the sections were stained with hematoxylin and eosin for histological examination under a light microscope.

### Cell culture and treatment

The human granulosa-like tumor cell line KGN was obtained from SUNNCELL (SNL−218, Shanghai, China). KGN cells were cultured in Dulbecco’s Modified Eagle Medium/Nutrient Mixture F−12 (DMEM/F−12; Thermo Fisher Scientific, USA), supplemented with 10% fetal bovine serum (FBS; Gibco, Life Technologies, USA) and 100 U/mL penicillin-streptomycin (Cytiva, China). Cells were maintained in a humidified incubator at 37 °C with 5% CO_2_ and passaged at 80–90% confluency.

For inhibitor experiments, cells were pretreated for 24 h with a STAT3 inhibitor, C188−9 (30 µM), an NLRP3 inhibitor, MCC950 (10 nM), or an AR inhibitor, Flutamide (1 µM). All inhibitors were purchased from Shanghai Yuanye Bio-Technology (Shanghai, China) and were dissolved in dimethyl sulfoxide (DMSO). The vehicle control group was treated with an equivalent volume of DMSO, with the final concentration maintained below 0.1% (v/v).

### Cell viability assay

Cell viability was evaluated using the Cell Counting Kit-8 (CCK-8; Bimake, Shanghai, China) following the manufacturer’s protocol. Briefly, KGN cells were seeded in 96-well plates at a density of 1 × 10⁴ cells/well. After overnight adherence, cells were treated with various concentrations of luteolin (0, 5, 10, and 25 µM) or dihydrotestosterone (DHT; 10 nM to 100 µM) for 24 h. Subsequently, 10 µL of CCK-8 solution was added to each well, followed by a 2-hour incubation at 37 °C. The absorbance was measured at 450 nm using a SpectraMax i3x microplate reader (Molecular Devices, San Jose, CA, USA). Cell viability was calculated as a percentage relative to the control group.

### LDH cytotoxicity assay

Cell membrane integrity was assessed by measuring lactate dehydrogenase (LDH) release into the culture medium using an LDH Cytotoxicity Assay Kit (Yuanye, R24020). KGN cells were seeded in 96-well plates at a density of 1 × 10⁴ cells/well and incubated for 12 h at 37 °C in a humidified incubator with 5% CO_2_. LDH levels were normalized to the control group, and all experiments were performed in triplicate.

### Immunohistochemistry (IHC)

Ovarian tissues were fixed, paraffin-embedded, and sectioned as previously described. Tissue sections were deparaffinized, rehydrated, and incubated with 3% H_2_O_2_ to quench endogenous peroxidase activity, followed by antigen retrieval using citrate buffer (Servicebio, China; G1202). After blocking with 5% goat serum for 1 h at room temperature, the sections were incubated overnight at 4 °C with primary antibodies against AR (1:1000, Abcam, America; ab133273), STAT3 (1:1000; Proteintech, China; 10253−2-AP), and NLRP3 (1:1000; Proteintech, China; 19771−1-AP). Sections were then incubated with HRP-conjugated secondary antibodies (1:2000, Servicebio, China; GB23303) at 37 °C for 1 h. Immunoreactivity was visualized using a DAB Detection Kit (Servicebio, China; G1212), and images were captured using an Olympus IX73 microscope (Olympus, Tokyo, Japan) and analyzed using ImageJ software to quantify the integrated optical density (IOD). For immunohistochemical analysis, ovarian tissues from three randomly selected rats per group were processed and quantified (*n* = 3).

### Immunofluorescence (IF)

Ovarian tissues and KGN cells were fixed with 4% paraformaldehyde and permeabilized using 0.5% Triton X−100 (Beyotime, Shanghai, China). After blocking with 5% bovine serum albumin for 1 h at room temperature, samples were incubated with primary antibodies against AR (1:1000; Abcam, ab133273), p-STAT3 (1:1000; Proteintech, 60479−1-Ig), STAT3 (1:1000; Proteintech, 10253−2-AP) and NLRP3 (1:1000; Proteintech, 19771−1-AP) at 4 °C overnight. After washing three times with PBS, samples were incubated with an appropriate fluorescent secondary antibody (1:1000; Beyotime, A0423) at room temperature for 1 h. Nuclei were counterstained with DAPI in the dark for 5 min. After mounting with an antifade reagent, fluorescence images were captured using a fluorescence microscope (Nikon, Tokyo, Japan). For both in vivo and in vitro experiments, three biological replicates were analyzed (*n* = 3). The mean fluorescence intensity (MFI) was quantified from multiple fields per sample using ImageJ software (National Institutes of Health, USA) to represent protein expression levels.

### Transmission electron microscopy (TEM)

For ultrastructural analysis, KGN cells were fixed in glutaraldehyde, post-fixed with osmium tetroxide, and then dehydrated in a graded series of ethanol. Subsequently, the samples were embedded in epoxy resin. Ultrathin sections were prepared using an ultramicrotome, stained with uranyl acetate and lead citrate, and examined with a transmission electron microscope (Hitachi H−7650, Japan). All experiments were performed in triplicate.

### Quantitative Real-Time PCR (qRT-PCR)

Total RNA was extracted from cells and ovarian tissues using TRIzol reagent (Invitrogen, USA). Reverse transcription was performed using 1 µg of RNA with the All Style Gold Reverse Transcription Kit (AE311−03, All Style). Quantitative real-time PCR was conducted using the TOLOBIO Amplification Kit on a real-time PCR system. Relative gene expression levels were calculated using the 2^^–ΔΔCt^ method, with GAPDH as the internal control. Primer sequences used in this study are listed in Table 1.

**Table 1:** The sequences of all primers used in this study

Gene	Species	Forward primer 5’→3’	Reverse primer 5’→3’
IL−1β	Rattus norvegicus	CAGCTTTCGACAGTGAGGAGA	TGTCGAGATGCTGCTGTGAG
IL−6	Rattus norvegicus	ACAAGTCCGGAGAGGAGACT	TTGCCATTGCACAACTCTTTTC
IL−18	Rattus norvegicus	TCAGACCACTTTGGCAGACT	GTCTGGGATTCGTTGGCTGT
NLRP3	Rattus norvegicus	CGGACTGACCCATCAATGCT	GCAGCTGACCAACCAGAGTT
GAPDH	Rattus norvegicus	CACCATCTTCCAGGAGCGAG	CTCGTGGTTCACACCCATCA
IL−6	Human	GCCACTCACCTCTTCAGAACGA	TCACCAGGCAAGTCTCCTCATT
IL−1	Human	TACCTGTCCTGCGTGTTGAAA	GGTGCTGATGTACCAGTTGGG
NLRP3	Human	GCGATCAACAGGAGAGACCTT	TCCACTCCTCTTCAATGCTGT
GAPDH	Human	GGAAGCTTGTCATCAATGGAAATC	TGATGACCCTTTTGGCTCCC

### Western blotting

Total protein was extracted from ovarian tissues and KGN cells using the Minute™ Total Protein Extraction Kit (Invent, USA). Protein concentrations were determined with the BCA Protein Assay Kit (Thermo Fisher Scientific, USA). Equal amounts of protein (approximately 20 µg) were separated by 10% SDS-PAGE and transferred onto PVDF membranes. Membranes were blocked with 5% non-fat milk at room temperature for 1 h and incubated overnight at 4 °C with primary antibodies. After washing, membranes were incubated with HRP-conjugated secondary antibodies, including Goat Anti-Rabbit IgG (1:5000; ZSGB-BIO, ZB−2301) or Goat Anti-Mouse IgG (1:5000; ZSGB-BIO, ZB−2305), for 2 h at room temperature. Protein bands were visualized using an enhanced chemiluminescence detection system. The relative protein expression levels were quantified using ImageJ software (National Institutes of Health, USA) and normalized to GAPDH. All experiments were performed with three biological replicates (*n* = 6).

The following primary antibodies were used: AR (1:1000; Abcam, ab133273), p-STAT3 (1:1000; Abcam, ab307602), NLRP3 (1:1000; Abcam, ab263899), Caspase−1 (1:1000; Abcam, ab272649), GSDMD (1:1000; Abcam, ab198029), IL−1β (1:1000; Abcam, ab315084), and GAPDH (1:1000; Proteintech, 60004−1-AP).

### Network Pharmacology analysis

Potential LUT targets were retrieved from TCMSP (Traditional Chinese Medicine Systems Pharmacology Database, https://www.tcmsp-e.com/), SwissTargetPrediction (http://swisstargetprediction.ch/), PubChem (https://pubchem.ncbi.nlm.nih.gov/). PCOS-related genes were obtained from OMIM (Online Mendelian Inheritance in Man, https://www.omim.org/) and DrugBank (https://go.drugbank.com/). Venn diagram analysis (BioVenn tool) was used to identify the intersection of targets between LUT and PCOS granulosa cell-related genes, which were considered potential therapeutic targets. Functional enrichment analysis was performed using DAVID (https://david.ncifcrf.gov/) for Gene Ontology (GO) terms (biological process, cellular component, molecular function) and KEGG Mapper (https://www.kegg.jp/kegg/mapper.html) for pathway analysis. The STRING database (https://string-db.org/) was used to generate a PPI network (confidence score > 0.7). Hub genes were ranked by degree centrality using Cytoscape (v3.9.1).

### Molecular Docking

Molecular docking simulations were performed using Dockey v1.0.3 software. The LUT was docked with the STAT3 protein (PDB ID 6NJS), IL−6 protein (PDB ID 4CNI), IL−1β (PDB ID 2NVH), and TNF protein (PDB ID 1A8M). Protein structures were retrieved from the Protein Data Bank (PDB) database (https://www.rcsb.org).

### Statistical analysis

Data are presented as mean ± standard error of the mean (SEM). All experiments were independently repeated at least three times. Statistical differences between two groups were evaluated using unpaired Student’s t-tests, while comparisons among multiple groups were conducted using one-way analysis of variance (ANOVA) followed by appropriate post hoc tests. Statistical analyses were performed using GraphPad Prism 9 (GraphPad Software, CA, USA). A p-value < 0.05 was considered statistically significant.

## Results

### Luteolin ameliorates endocrine, ovarian, and inflammatory pathologies in PCOS rats

**Fig. 1:**
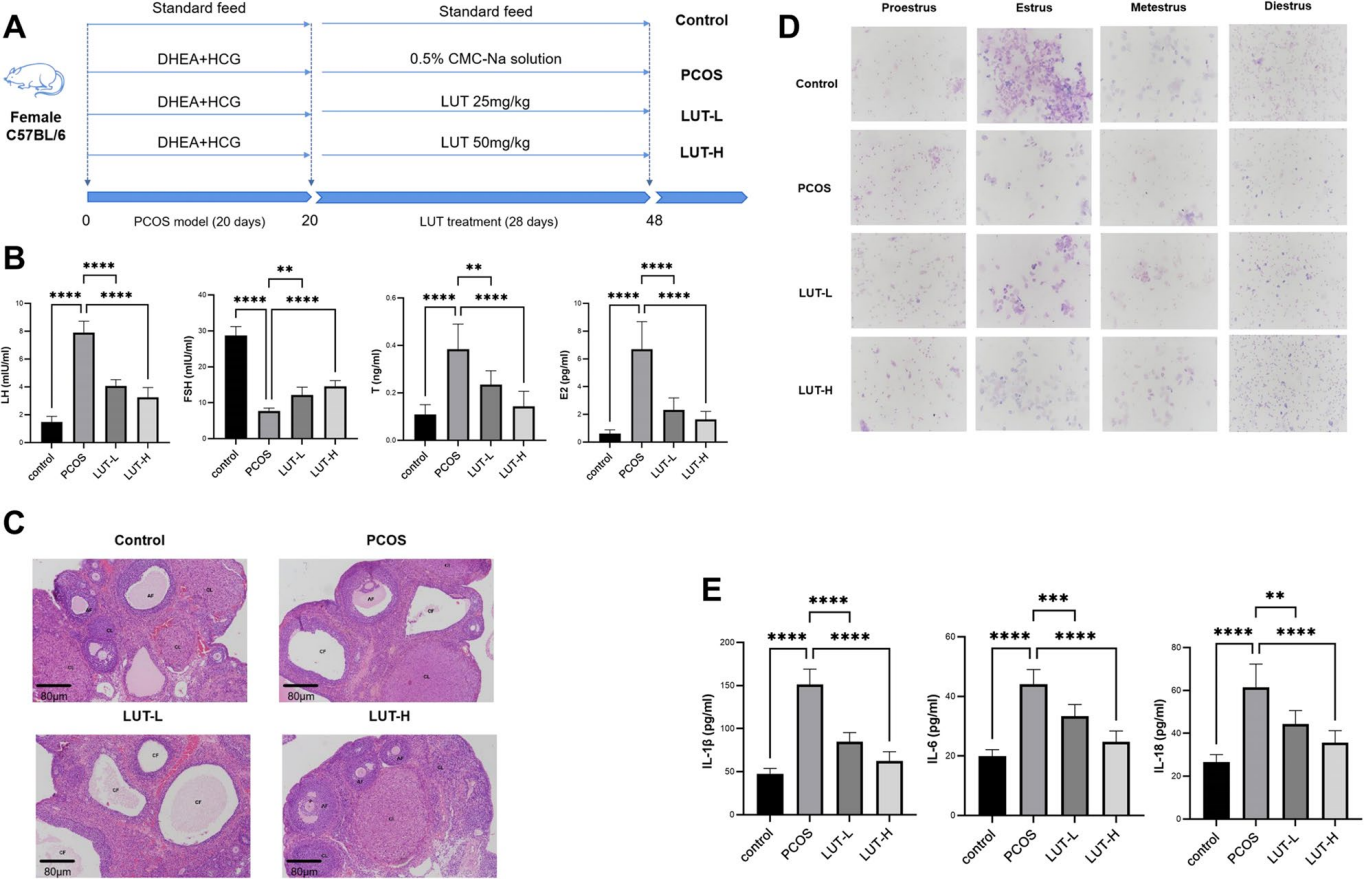
Luteolin ameliorates endocrine, ovarian, and inflammatory pathologies in PCOS rats. **A** Schematic diagram of the experimental design, including DHEA-induced PCOS modeling and LUT treatment. **B** Serum levels of LH, FSH, T and E2 measured by ELISA. **C** Representative H&E-stained ovarian sections from each group. Scale bar = 80 μm. **D** Representative images of vaginal smears showing distinct phases of the estrous cycle. **E** Serum levels of inflammatory factors IL-6, IL-1β and IL-18 by ELISA in rats from different groups. Data are presented as mean ± SEM (*n* = 6 per group). Statistical significance was determined by one-way ANOVA with Tukey’s post-hoc test. **p < 0.01, ***p < 0.001, ****p < 0.0001 compared to the PCOS model group.

The therapeutic potential of two LUT dosages, 25 mg/kg (LUT-L) and 50 mg/kg (LUT-H), was evaluated in a rat model of PCOS, based on previous reports [19, 20] (Fig. 1A). The PCOS group displayed typical endocrine disruptions, including decreased FSH alongside elevated LH, T, and E2. LUT treatment dose-dependently corrected these hormonal imbalances (Fig. 1B). Histologically, ovaries from PCOS rats showed cystic follicles and fewer corpora lutea, abnormalities that were markedly ameliorated by LUT (Fig. 1C). Correspondingly, LUT administration substantially corrected the irregular estrous cycles, indicating a partial restoration of follicular development and ovulation (Fig. 1D). The PCOS-induced systemic inflammation, evidenced by elevated serum IL-6, IL-1β, and IL-18, was also robustly suppressed by LUT (Fig. 1E). Collectively, these results indicate that luteolin effectively alleviates the endocrine, ovarian, and systemic inflammatory features of PCOS in a dose-dependent manner.

### Network Pharmacology reveals STAT3 as a key target of Luteolin in PCOS

**Fig. 2:**
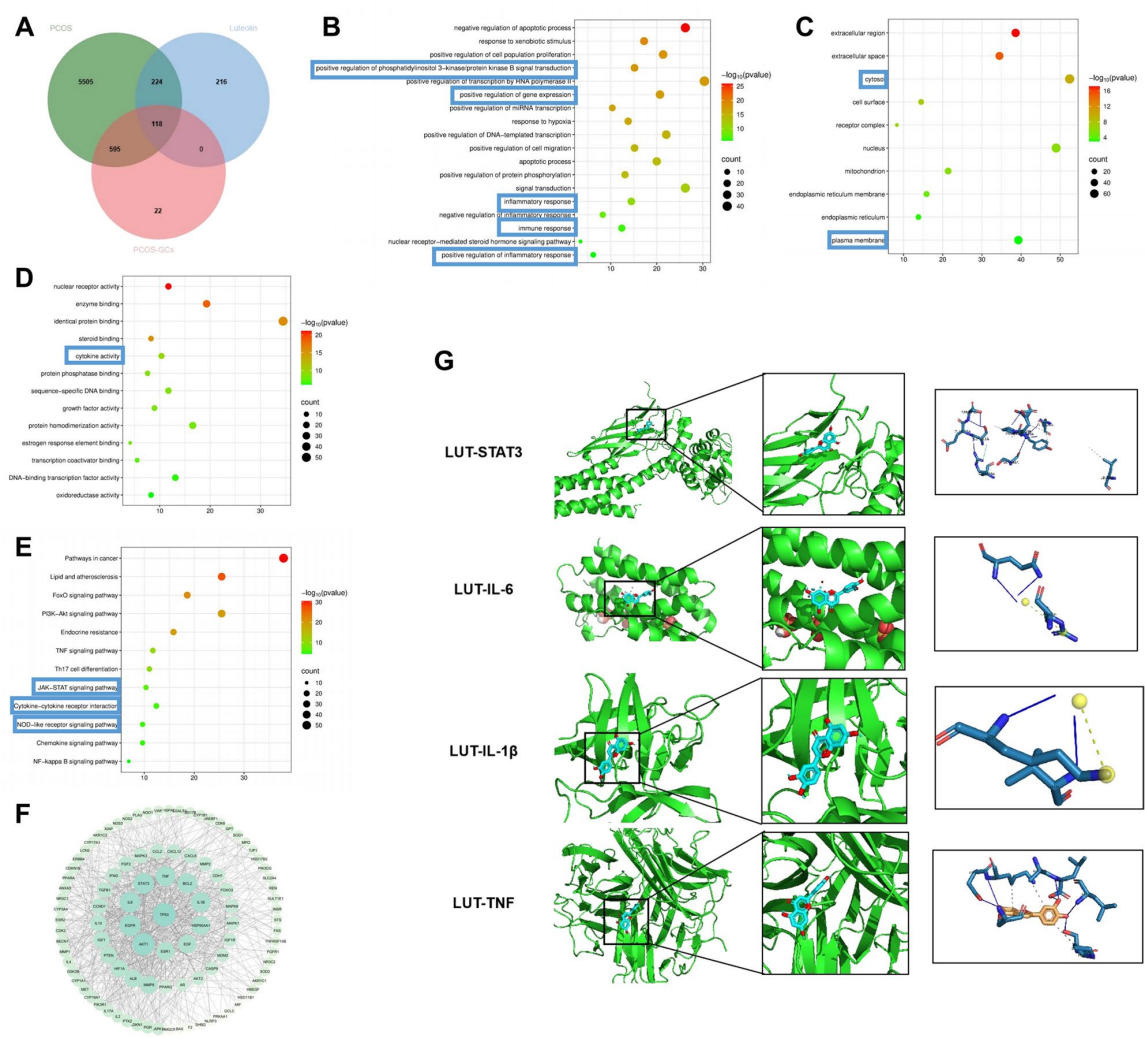
Network pharmacology reveals STAT3 as a key target of luteolin in PCOS. **A** Venn diagram showing the overlap of targets related to LUT, PCOS, and PCOS granulosa cells. **B**-**E** GO and KEGG enrichment analysis of the potential targets. **F** PPI network of the overlapping targets. **G** Molecular docking results showing the binding of luteolin (LUT) to hub targets STAT3, IL-6, IL-1β, and TNF.

To systematically explore the potential mechanisms of LUT in PCOS, a network pharmacology approach was employed (Fig. 2A). A total of 558 potential LUT targets were collected from public databases (TCMSP, SwissTargetPrediction, PubChem), while 6,442 PCOS-related genes and 735 granulosa cell-specific genes were retrieved from disease and expression databases (OMIM, DrugBank). Cross-analysis using Venn diagrams identified 118 overlapping genes, which may serve as LUT-responsive targets involved in granulosa cell regulation in PCOS.

Gene Ontology (GO) and Kyoto Encyclopedia of Genes and Genomes (KEGG) enrichment analyses indicated that the overlapping genes were significantly enriched in inflammation-related pathways, particularly the JAK-STAT signaling and NOD-like receptor signaling pathways (Fig. 2B–E). These findings suggest a potential role of LUT in modulating immune-inflammatory responses in the ovarian microenvironment. Subsequently, a protein–protein interaction (PPI) network was constructed using the STRING database and visualized with Cytoscape. Topological analysis based on degree centrality identified STAT3, Tumor Protein P53 (TP53), Epidermal Growth Factor Receptor (EGFR), IL-6, TNF, and IL-1β as major hub genes (Fig. 2F). Although TP53 and EGFR showed high connectivity, they were deprioritized due to their limited direct relevance to pyroptosis in granulosa cells. Therefore, STAT3, IL-6, IL-1β, and TNF were selected for molecular docking to assess their interaction with LUT. Among these candidates, STAT3 exhibited the strongest binding affinity with LUT (–8.589 kcal/mol; PDB ID: 6NJS), suggesting a stable interaction (Fig. 2G; Table 2). Considering its well-established role in activating the NLRP3 inflammasome and promoting pyroptosis, STAT3 was identified as a key inflammation-related target of LUT in PCOS.

Based on these findings, the STAT3–NLRP3 axis was prioritized for further mechanistic validation in subsequent in vitro and in vivo experiments.

**Table 2:** Binding affinities of Luteolin with PCOS-related hub genes predicted by molecular Docking

Hub gene	PDB ID	Affinity (kcal/mol)
STAT3	6NJS	−8.589
IL−6	2NVH	−8.173
IL−18	1ALU	−7.322
TNF	1A8M	−8.029

### Luteolin inhibits the expression of STAT3 and NLRP3 and attenuates granulosa cell pyroptosis in PCOS rats

**Fig. 3:**
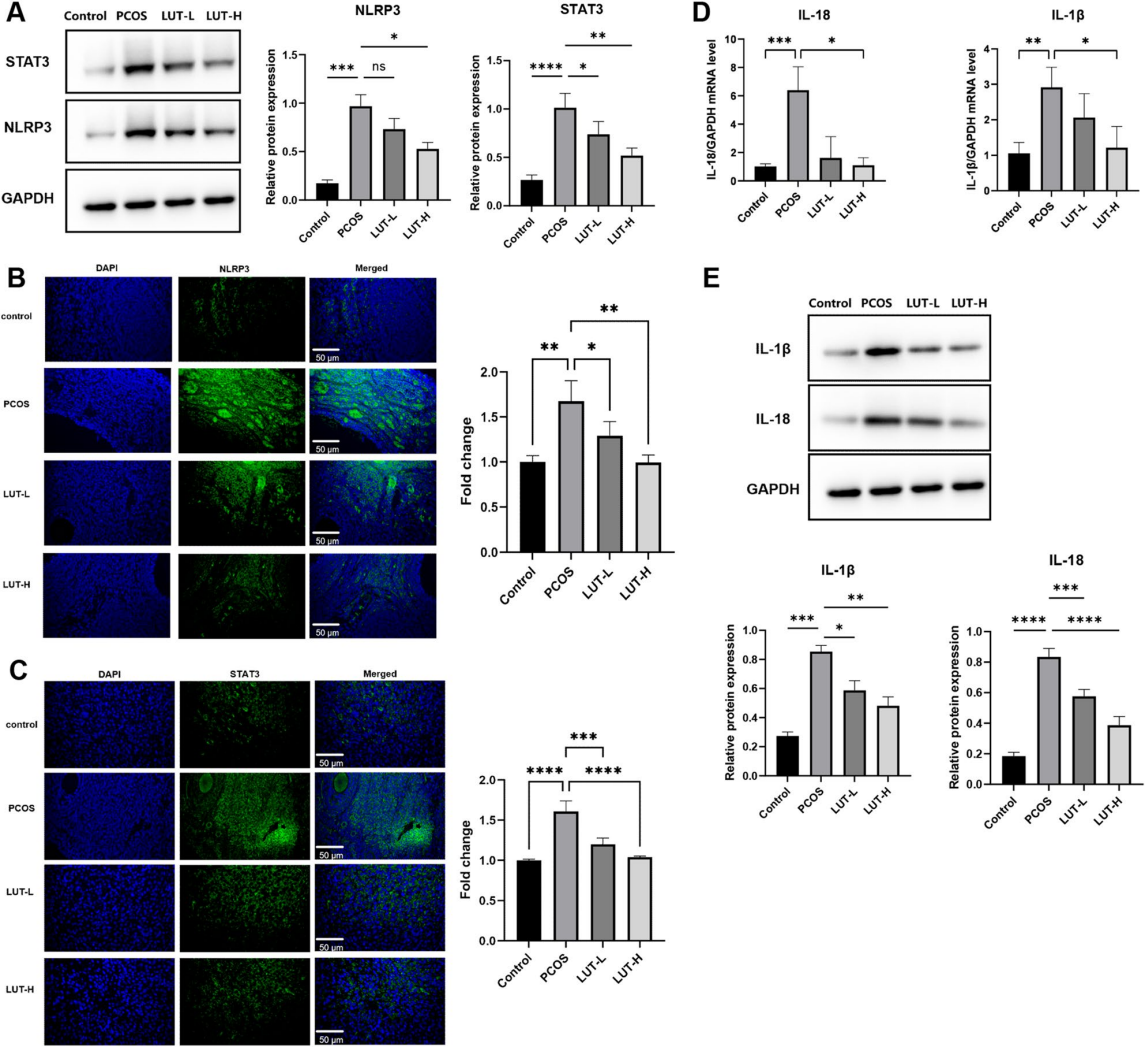
Luteolin inhibits the expression of STAT3 and NLRP3 and attenuates granulosa cell pyroptosis in PCOS rats. **A** Western blot analysis and quantification of STAT3 and NLRP3 protein levels in each group (*n* = 6 per group). **B**-**C** Immunofluorescence staining showing the expression of STAT3 and NLRP3 (*n* = 3 per group). Scale bar = 50 μm (400×). **D** qRT-PCR analysis of IL-18 and IL-1β mRNA levels (*n* = 6 per group). **E** Western blot analysis of IL-18 and IL-1β protein levels (*n* = 6 per group). All experiments were performed in triplicate. Data are presented as mean ± SEM. Statistical significance was determined by one-way ANOVA with Tukey’s post-hoc test. *p < 0.05, **p < 0.01, ***p < 0.001, ****p < 0.0001 compared to the DHT-treated model group.

To investigate the involvement of the key signaling molecules STAT3 and NLRP3, which were predicted by our initial analysis, we examined their expression in ovarian tissues. Western blot analysis revealed significant upregulation of STAT3 (*p* < 0.001) and NLRP3 (*p* < 0.001) in PCOS rats compared to controls. Notably, treatment with LUT resulted in a dose-dependent suppression of both proteins, with LUT-H producing a statistically significant reduction in both STAT3 (*p* < 0.01) and NLRP3 (*p* < 0.05) (Fig. 3A). The LUT-L showed a downward trend in NLRP3 expression, although the change did not reach statistical significance (*p* > 0.05).

These results were further confirmed by immunofluorescence staining and subsequent quantification of fluorescence intensity. Granulosa cells from PCOS rats exhibited significantly stronger cytoplasmic signals for both STAT3 (*p* < 0.0001) and NLRP3 (*p* < 0.01) compared to the control group. Consistent with the Western blot data, LUT-H treatment markedly reduced the fluorescence intensity of both STAT3 (*p* < 0.0001) and NLRP3 (*p* < 0.01) relative to the PCOS group (Fig. 3B-C). Given the central roles of both STAT3 and NLRP3 in inflammation and pyroptosis, we next assessed downstream inflammatory mediators. At both the mRNA and protein levels, the key pyroptotic cytokines IL-1β and IL-18 were significantly elevated in the ovaries of PCOS rats compared to controls (*p* < 0.05 for all comparisons). Luteolin administration significantly suppressed the expression of these markers, particularly in the LUT-H group (Fig. 3D–E), indicating an effective suppression of the pyroptosis-associated inflammatory response.

Taken together, these results demonstrate that luteolin alleviates granulosa cell pyroptosis in PCOS. This therapeutic effect is strongly associated with the downregulation of two key signaling proteins, STAT3 and NLRP3, and the subsequent inhibition of their downstream inflammatory cascade.

### Luteolin alleviates DHT-induced pyroptosis in human granulosa cells in vitro

**Fig. 4:**
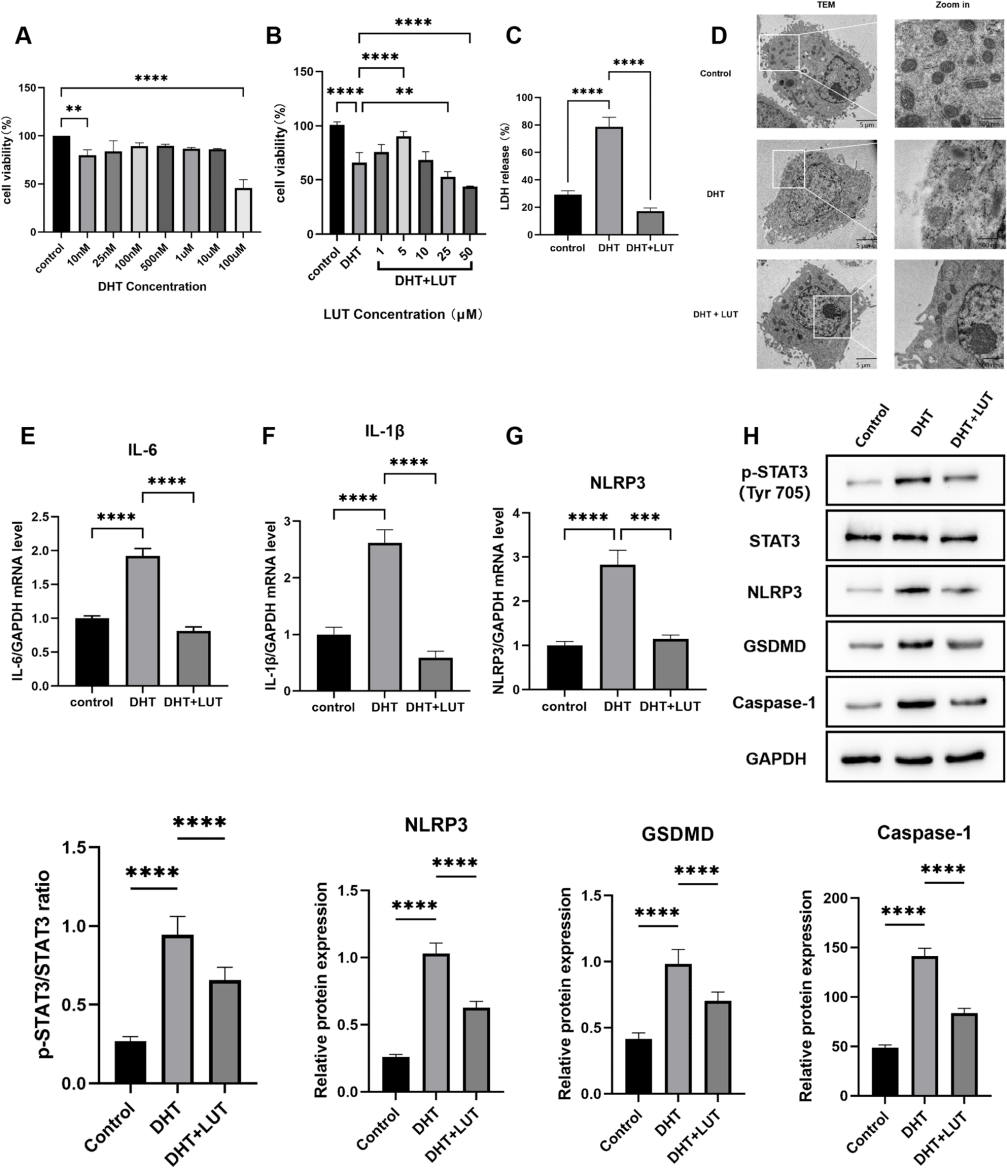
Luteolin alleviates DHT-induced pyroptosis in human granulosa cells in vitro. **A**-**B** Cell viability assessed by CCK8 assay at various concentrations of (**A**) DHT and (**B**) LUT. **C** LDH release assay. **D** Representative transmission electron microscopy (TEM) images displaying cellular ultrastructure. **E**–**G** qRT-PCR analysis of IL-6, IL-1β, and NLRP3 mRNA levels in different treatment groups. **H** Western blot analysis of p-STAT3, STAT3, NLRP3, GSDMD, and Caspase-1 protein levels. Data are presented as mean ± SEM from three independent experiments (*n* = 3). Statistical significance was determined by one-way ANOVA with Tukey’s post-hoc test. *p < 0.05, **p < 0.01, ***p < 0.001, ****p < 0.0001 compared to the DHT-treated group.

To establish a cell injury model, we first determined a suitable concentration of DHT. KGN cells were treated with DHT at concentrations ranging from 10 nM to 100 µM for 24 h. The CCK-8 assay revealed that KGN cell viability was significantly reduced upon treatment with 10 nM and 100 µM DHT (Fig. 4A). Specifically, 10 nM DHT decreased viability to approximately 80% (*p* < 0.01), while the 100 µM concentration caused a more severe reduction to below 50%. Consequently, 10 nM DHT was used in all subsequent experiments.

Next, we investigated the protective effect of LUT against DHT-induced injury. Co-treatment with 5 µM LUT, a non-cytotoxic concentration determined in preliminary assays, restored the viability of DHT-treated cells (*p* < 0.0001) (Fig. 4B). Consistent with this, the elevated LDH release in DHT-treated cells, a marker of membrane damage, was suppressed by the addition of LUT (*p* < 0.0001) (Fig. 4C). To further evaluate cell death morphology, TEM revealed classical features of pyroptosis in DHT-treated cells, including cytoplasmic swelling, membrane blebbing, organelle disruption, and mitochondrial fragmentation. In contrast, LUT treatment preserved membrane integrity and mitochondrial ultrastructure (Fig. 4D).

At the molecular level, RT-qPCR analysis showed that DHT significantly upregulated NLRP3 (*p* < 0.001), IL-6 (*p* < 0.0001), and IL-1β (*p* < 0.0001) mRNA expression, all of which were downregulated following LUT treatment (Fig. 4E–G). Western blot analysis further showed that DHT promoted phosphorylated STAT3 (p-STAT3), NLRP3 expression, and the activation of caspase-1, GSDMD, and IL-1β (all *p* < 0.0001). These effects were substantially reversed by LUT co-treatment (all *p* < 0.0001) (Fig. 4H). Taken together, these results demonstrate that LUT protects granulosa cells from DHT-induced pyroptosis. This protective effect appears to be mediated by attenuating the activation of the STAT3-NLRP3 signaling pathway.

### Pharmacological Inhibition of STAT3 mimics the Anti-pyroptotic effect of Luteolin

**Fig. 5:**
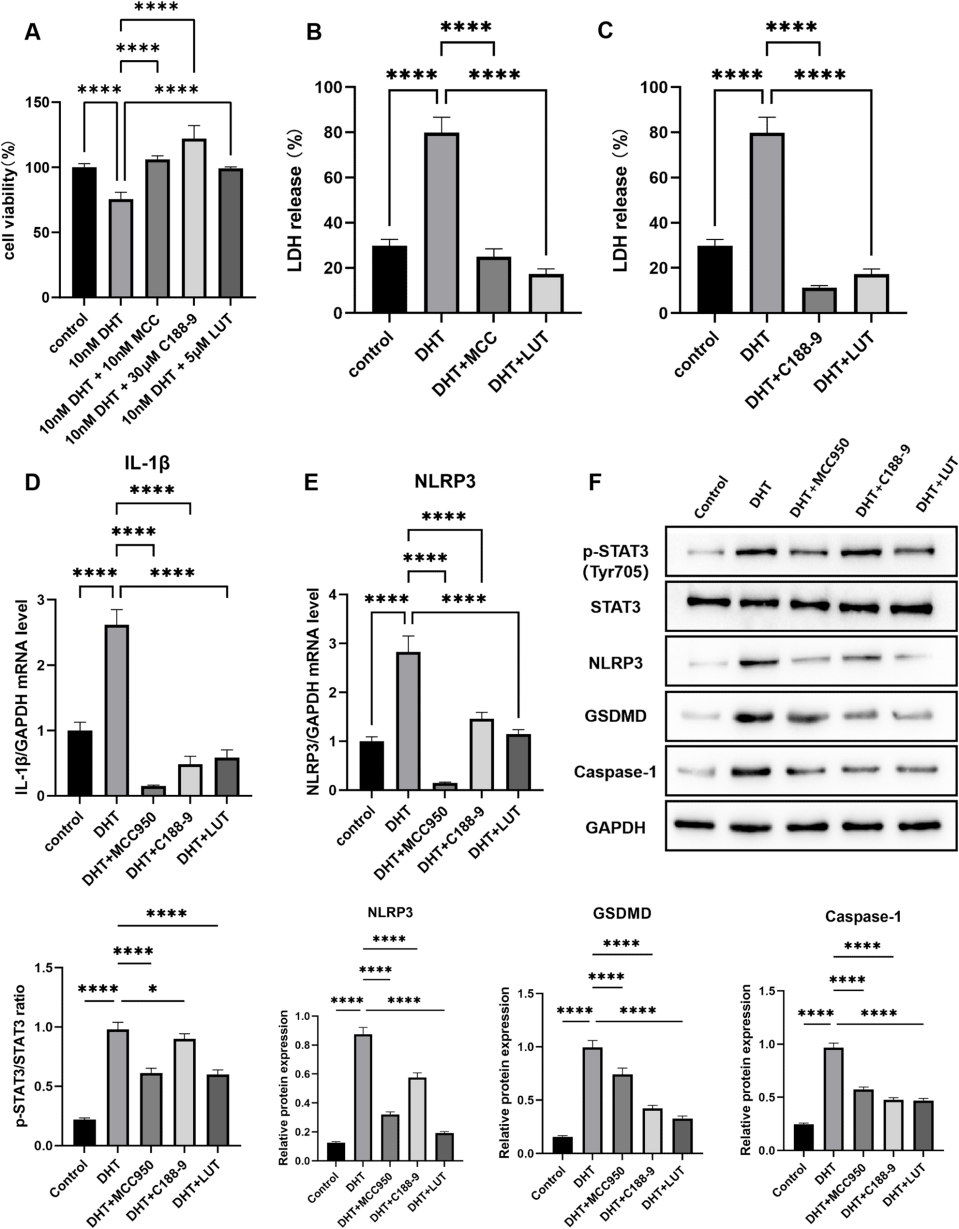
Pharmacological Inhibition of STAT3 Mimics the Anti-pyroptotic Effect of Luteolin. **A** CCK8 assay for determining the cytotoxicity of MCC and C188-9. **B**-**C** LDH release levels in different groups. **D**-**E** qRT-PCR analysis of IL-1β and NLRP3 mRNA levels. **F** Western blot analysis of p-STAT3, STAT3, NLRP3, GSDMD, and Caspase-1 protein levels. Data are presented as mean ± SEM from three independent experiments. Statistical significance was determined by one-way ANOVA with Tukey’s post-hoc test. *p < 0.05, **p < 0.01, ***p < 0.001, ****p < 0.0001 compared to the DHT-treated group.

To elucidate the mechanism by which LUT inhibits pyroptosis, we utilized pharmacological inhibitors targeting STAT3 (C188-9) and NLRP3 (MCC950). First, dose-response curves were established to determine the optimal non-toxic working concentrations of C188-9 (30 µM) and MCC950 (10 nM) in KGN cells (Fig. 5A). Next, we compared the effects of these inhibitors with LUT in DHT-treated cells. As shown by an LDH release assay, DHT treatment significantly increased LDH leakage compared to the control group (*p* < 0.0001). This increase was significantly reversed by the addition of LUT, C188-9, or MCC950 (all *p* < 0.0001 vs. DHT group) (Fig. 5B-C). Similarly, qRT-PCR analysis demonstrated that the DHT-induced upregulation of IL-1β and NLRP3 mRNA was significantly suppressed by all three treatments (*p* < 0.0001 for all comparisons against the DHT group) (Fig. 5D–E). To further dissect the pathway, we examined protein levels via Western blot. Consistent with our hypothesis, both LUT and the STAT3 inhibitor C188-9 significantly reduced the DHT-induced phosphorylation of STAT3. This was accompanied by a significant downstream reduction in the protein levels of NLRP3, Caspase-1, and cleaved IL-1β (Fig. 5F). Interestingly, the NLRP3 inhibitor MCC950 not only suppressed its target and downstream pyroptotic markers but also led to a significant reduction in p-STAT3, an effect comparable to that of C188-9.

### Luteolin ameliorates hyperandrogen-induced pyroptosis by inhibiting the AR/STAT3/NLRP3 axis

**Fig. 6:**
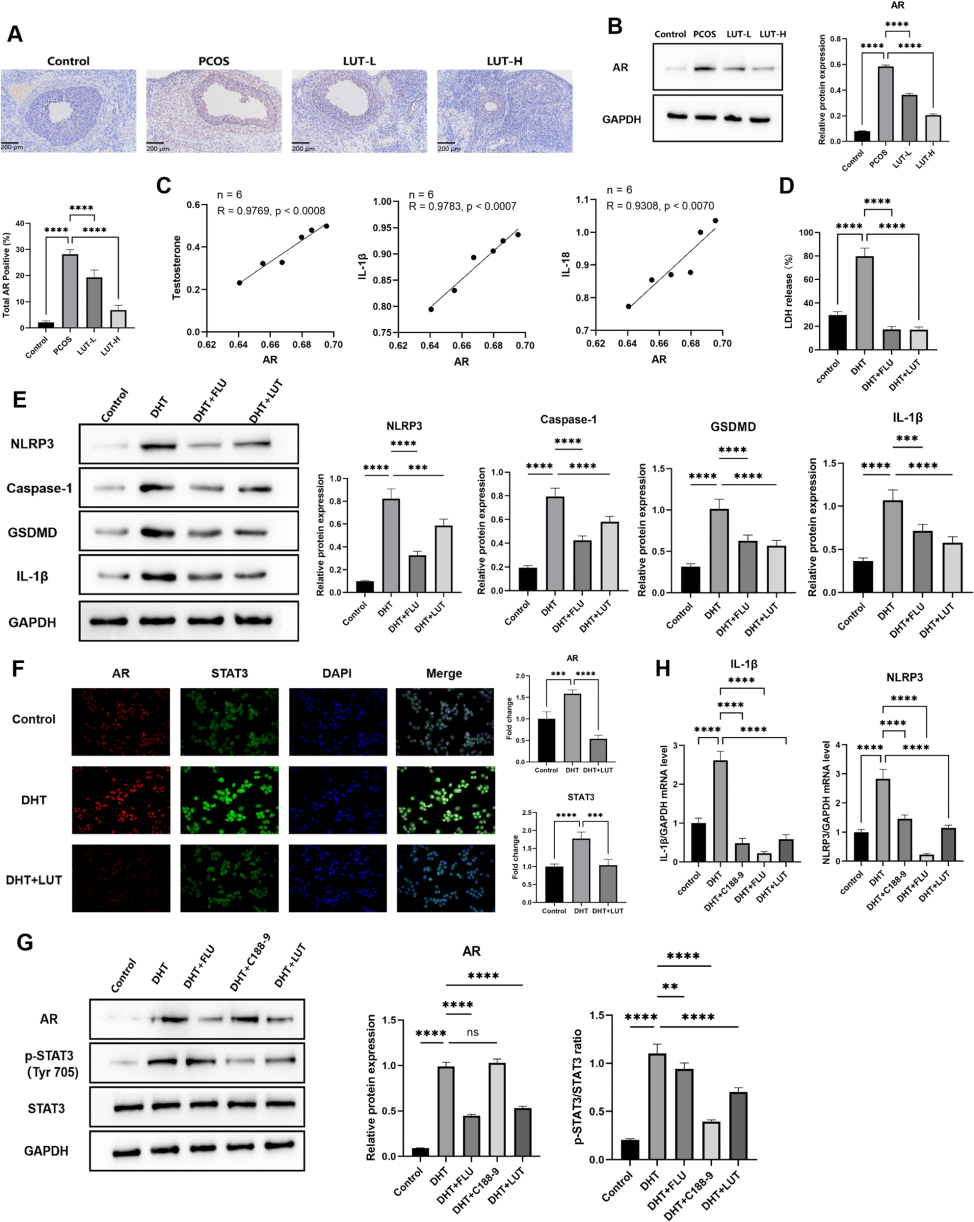
Luteolin ameliorates hyperandrogen-induced pyroptosis by inhibiting the AR/STAT3/NLRP3 axis. **A** Representative immunohistochemical staining of AR in rat ovarian tissue (*n* = 6 per group). Scale bar = 200 μm. **B** Western blot analysis and quantification of AR protein levels (*n* = 6 per group). **C** Correlation analysis among AR, testosterone, IL-1β, and IL-18 (*n* = 6 per group). **D** LDH release levels (*n* = 3 independent experiments). **E** Western blot analysis of NLRP3, GSDMD, and Caspase-1 protein levels (*n* = 3 independent experiments). **F** Immunofluorescence staining of AR and STAT3. Scale bar = 50 μm (400×) (*n* = 3 per group). **G** Western blot analysis of AR, p-STAT3, and STAT3 (*n* = 3 independent experiments). (H) qRT-PCR analysis of IL-1β and NLRP3 mRNA levels in KGN cells (*n* = 3 independent experiments). Data are presented as mean ± SEM. Statistical significance was determined by one-way ANOVA with Tukey’s post-hoc test. *p < 0.05, **p < 0.01, ***p < 0.001, ****p < 0.0001.

In our PCOS rat model, immunohistochemistry and Western blot analyses revealed a significant increase in AR expression, which was dose-dependently suppressed by LUT treatment (*p* < 0.0001) (Fig. 6A–B). Correlation analysis, performed using data from individual animals exclusively within the PCOS model group (*n* = 6), demonstrated a strong positive association between AR expression and serum testosterone levels (*r* = 0.9769), as well as with the pro-inflammatory cytokines IL-1β (*r* = 0.9783) and IL-18 (*r* = 0.9308) (Fig. 6C). To determine if AR signaling directly mediates granulosa cell pyroptosis, DHT-induced KGN cells were treated with the AR inhibitor flutamide. This treatment (1 µM) significantly reduced LDH release (*p* < 0.0001) (Fig. 6D). Consistent with this, Western blot analysis showed that flutamide suppressed the expression of pyroptosis-related proteins, including NLRP3, Caspase-1, GSDMD, and cleaved IL-1β (*p* < 0.0001) (Fig. 6E and supplement Fig. 1A).

Next, we investigated the link between AR and STAT3 signaling. Immunofluorescence analysis revealed that DHT induced nuclear translocation of both AR and p-STAT3, which was markedly reversed by LUT co-treatment (Fig. 6F). To establish the hierarchy of this pathway, we performed mechanistic inhibitor studies. Western blot analysis showed that flutamide downregulated both AR (*p* < 0.0001) and p-STAT3 (*p* < 0.01). Conversely, the STAT3-specific inhibitor C188-9 suppressed p-STAT3 (*p* < 0.0001) without affecting AR levels (*p* > 0.05), positioning AR upstream of STAT3 (Fig. 6G).

Finally, qRT-PCR analysis showed that LUT, flutamide, and C188-9 all significantly downregulated NLRP3 and IL-1β mRNA expression in DHT-treated cells (*p* < 0.0001) (Fig. 6H). Taken together, these data suggest that luteolin ameliorates hyperandrogen-induced pyroptosis in granulosa cells by inhibiting a signaling cascade involving AR, STAT3, and the subsequent activation of the NLRP3 inflammasome.

## Discussion

PCOS is an endocrine-metabolic disorder defined by hyperandrogenism, ovulatory dysfunction, and chronic low-grade inflammation. This study reveals identifies the AR/STAT3/NLRP3 signaling axis as a critical driver of granulosa cell pyroptosis in response to hyperandrogenism. We further demonstrate that LUT exerts its therapeutic effects by specifically targeting this pathway, thereby attenuating ovarian inflammation and dysfunction in a DHEA-induced rat model of PCOS.

Our study identifies the AR as a key initiator of this inflammatory cascade. In PCOS rats ovaries, we found that elevated AR expression strongly correlated with serum testosterone and inflammatory cytokines, underscoring its fundamental role in the local inflammatory process. This was further supported by immunofluorescence analysis, which revealed nuclear co-localization of AR and p-STAT3 in DHT-treated granulosa cells. Mechanistically, we established a clear upstream function for AR, as its pharmacological inhibition suppressed p-STAT3 expression, whereas STAT3 inhibition did not alter AR levels. These results provide a direct link between androgen excess and the activation of the key inflammatory regulator STAT3.

Downstream of the AR/STAT3 activation, our study validates the NLRP3 inflammasome as central effector of pyroptosis in hyperandrogenic granulosa cells. Our initial network pharmacology and molecular docking analyses predicted a strong binding affinity between luteolin and key pathway proteins, predictions that were subsequently validated by our experimental data. Crucially, these in silico predictions were subsequently validated by our in vivo and in vitro experiments. We confirmed that STAT3 inhibition downregulated NLRP3 expression, positioning NLRP3 downstream in the signaling cascade. Intriguingly, pharmacological inhibition of NLRP3 with MCC950 also suppressed p-STAT3, implying a potential crosstalk or positive feedback mechanism between the two pathways [23, 24].

Having elucidated this pathological pathway, we identified LUT as a potent inhibitor. In our DHEA-induced PCOS rat model, LUT’s ability to suppress the AR/STAT3/NLRP3 axis resulted in significant physiological improvements. Specifically, LUT treatment ameliorated endocrine abnormalities, evidenced by reduced serum T and LH levels, elevated FSH, and restored estrous cycle regularity. While prior studies have highlighted LUT’s role in regulating steroidogenesis and insulin sensitization [19, 20], our work reveals a novel anti-inflammatory function for LUT, mediated by its ability to directly inhibit androgen-driven pyroptosis in the ovary. Our findings reinforce the therapeutic potential of targeting the NLRP3 inflammasome in PCOS. For instance, a recent study demonstrated that carvacrol, another natural compound, ameliorated PCOS symptoms in a rat model. This therapeutic effect was associated with its potent antioxidative properties and its ability to inhibit the NLRP3/NF-κB signaling pathway [25], which strongly supports our focus on the NLRP3 inflammasome as a key target. The convergence of findings from different PCOS models and with different therapeutic agents strongly underscores the central role of the NLRP3 inflammasome in the pathogenesis of PCOS.

Despite these significant findings, this study has several limitations. First, our preclinical models have translational constraints. The DHEA-induced rat model, while recapitulating hyperandrogenism, represents a lean, non-insulin-resistant PCOS phenotype [26], which does not capture the metabolic heterogeneity of human PCOS. Future studies in diet-induced obesity models are needed. Similarly, the KGN cell line may not fully reflect the physiology of primary granulosa cells, and the absence of validation in human clinical samples limits the direct applicability of our findings. Future studies are therefore planned to validate these findings by examining the AR/STAT3/NLRP3 axis in granulosa cells collected from human PCOS patients. Second, the molecular interactions require deeper investigation. While the functional link between AR and STAT3 is established, it is considered indirect. Therefore, confirming a direct physical interaction requires further validation through co-immunoprecipitation (Co-IP) or chromatin immunoprecipitation (ChIP) assays. Furthermore, our correlation analysis linking AR expression to inflammatory markers, while statistically significant, was based on a small sample size (n = 6) and should be interpreted as preliminary evidence requiring confirmation in larger cohorts. Likewise, the predicted binding of luteolin to its targets requires biophysical confirmation via assays like Surface Plasmon Resonance (SPR). Finally, the therapeutic profile of luteolin requires further assessment. Although considered non-toxic, luteolin lacks ‘Generally Recognized as Safe’ (GRAS) status from the U.S. FDA, warranting further safety studies [27]. Moreover, its clinical translation is limited by poor oral bioavailability (~ 26% in rats) resulting from low solubility and significant first-pass metabolism [28]. Consequently, the effective doses from our animal model cannot be directly extrapolated to humans, necessitating future work on optimized delivery systems. This assessment must also include potential off-target effects and the interplay between its anti-pyroptotic and known metabolic benefits. Methodologically, we also acknowledge that the lack of blinding during data evaluation is a limitation that should be addressed in future work.

## Conclusion

In conclusion, our study defines a novel AR/STAT3/NLRP3 signaling axis that drives granulosa cell pyroptosis in PCOS. We identify luteolin as a promising therapeutic agent that effectively targets this axis. These findings provide a robust mechanistic foundation and highlight a promising strategy for therapeutic intervention in PCOS.

## Supplementary Information

Supplementary Material 1

## Data Availability

The datasets generated and/or analysed during the current study are available from the following sources:1. Publicly available data: The data supporting the network pharmacology analysis were obtained from public databases, including TCMSP, SwissTargetPrediction, PubChem, OMIM, and DrugBank. All web links and access methods are provided in the Methods section of this manuscript.2. Original experimental data: The original datasets generated and analysed during the animal and cell studies are not publicly available due to their large size but are available from the corresponding author on reasonable request.

## Abbreviations

PCOS: Polycystic ovary syndrome
LUT: Luteolin
DHEA: Dehydroepiandrosterone
ELISA: Enzyme-linked immunosorbent assay
DHT: Dihydrotestosterone
CCK−8: Cell Counting Kit−8
LDH: Lactate dehydrogenase
AR: Androgen receptor
STAT3: Signal Transducer and Activator of Transcription 3
NLRP3: NOD-like Receptor Pyrin domain-containing protein 3
HA: Hyperandrogenemia
IL-1β: Interleukin-1β
IL-18: Interleukin-18
SD: Sprague–Dawley
SPF: specific pathogen-free
HCG: human chorionic gonadotropin
CMC-Na: carboxymethylcellulose sodium
FSH: follicle-stimulating hormone
LH: luteinizing hormone
T: testosterone
E2: estradiol
IL−6: interleukin−6
H&E: Hematoxylin and eosin staining
DMEM/F−12: Dulbecco’s Modified Eagle Medium/Nutrient Mixture F−12
FBS: fetal bovine serum
DMSO: dissolved in dimethyl sulfoxide
OD: optical density
IOD: integrated optical density
IF: Immunofluorescence
MFI: mean fluorescence intensity
GO: Gene Ontology
KEGG: Kyoto Encyclopedia of Genes and Genomes
PPI: protein–protein interaction
TP53: Tumor Protein P53
EGFR: Epidermal Growth Factor Recepto
TEM: transmission electron microscopy
Co-IP: co-immunoprecipitation
ChIP: chromatin immunoprecipitation
SPR: Surface Plasmon Resonance
