# Supplementary figures and images for: Luteolin alleviates PCOS by inhibiting AR/STAT3/NLRP3-mediated granulosa cell pyroptosis
Source: J Ovarian Res. 2026 Jan 24;19:63. doi: 10.1186/s13048-025-01952-4 (PMC12911274; doi:10.1186/s13048-025-01952-4)

**A**

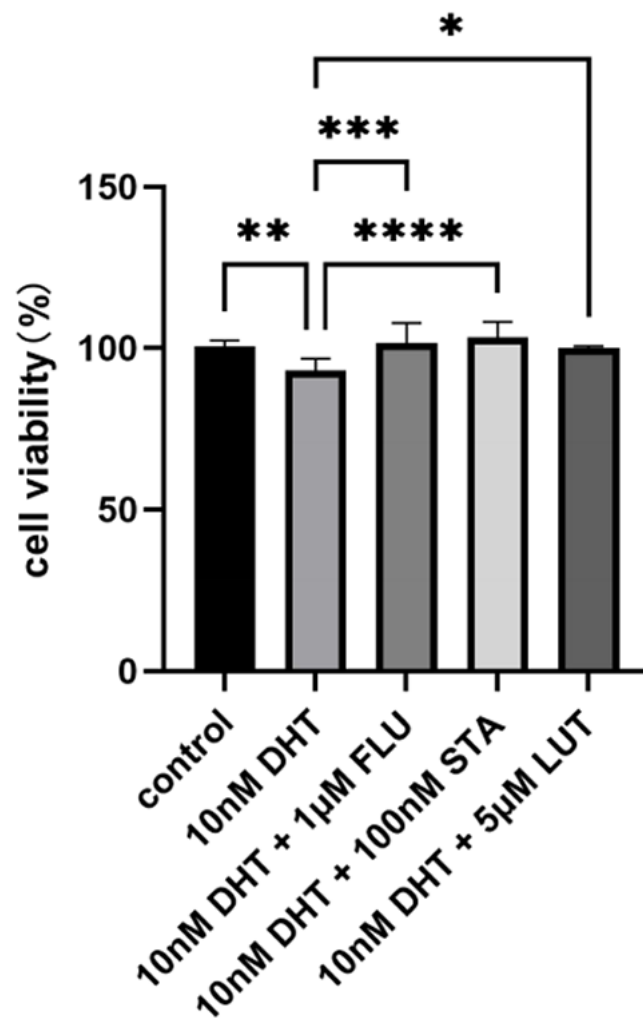

Supplement: Supplementary file 1 — Supplementary Material 1 [file 13048_2025_1952_MOESM1_ESM.pdf]
